# Clinical Impact of Infection with Pandemic Influenza (H1N1) 2009 Virus in Naïve Nucleus and Multiplier Pig Herds in Norway

**DOI:** 10.1155/2011/163745

**Published:** 2011-12-22

**Authors:** Carl Andreas Grøntvedt, Chiek Er, Britt Gjerset, Anna Germundsson, Tore Framstad, Edgar Brun, Anne Jørgensen, Bjørn Lium

**Affiliations:** ^1^Norwegian School of Veterinary Science, Department of Production Animal Clinical Sciences, P.O. Box 8146, 0033 Oslo, Norway; ^2^Department of Health Surveillance, Norwegian Veterinary Institute, P.O. Box 750, 0106 Oslo, Norway; ^3^Department of Laboratory Services, Norwegian Veterinary Institute, P.O. Box 750, 0106 Oslo, Norway; ^4^Norwegian Pig Health Service, Animalia, P.O. Box 396, 0513 Oslo, Norway

## Abstract

The Norwegian pig population has been free from influenza viruses until 2009. The pandemic influenza outbreak during the autumn 2009 provided an opportunity to study the clinical impact of this infection in an entirely naïve pig population. This paper describes the results of a case-control study on the clinical impact of pandemic influenza (H1N1) 2009 infection in the nucleus and multiplier herds in Norway. The infection spread readily and led to seroconversion of 42% of the Norwegian nucleus and multiplier herds within a year. Positive and negative herds were identified based on surveillance data from the Norwegian Veterinary Institute. Telephone interviews were conducted with pig herd owners or managers between November 2010 and January 2011. Pigs with clinical signs were reported from 40% of the case herds with varying morbidity and duration of respiratory disease and reduced reproductive performance. Clinical signs were reported in all age groups.

## 1. Introduction

 Pandemic influenza A (H1N1) 2009 virus was first recorded in Norwegian pig herds in October 2009 [1]. Before that, documentation on freedom from several specific viral diseases in the pig population was provided by the surveillance and control program, where swine influenza (subtypes H1N1 and H3N2) has been included since 1997 [2]. All the nucleus and multiplying herds are included in this program.

The Norwegian pig population is also documented free from porcine reproductive and respiratory syndrome virus, Aujeszky's disease, porcine respiratory corona virus, and transmissible gastroenteritis [2]. In 2009 the pig population in Norway was declared free from enzootic pneumonia (*Mycoplasma hyopneumoniae*) [3]. Porcine circovirus type 2 is, however, presumed to be present in all Norwegian swine herds, including nucleus and multiplier herds.

In contrast to Norway, the pig populations in most other countries are endemically infected with different swine adapted subtypes of influenza A virus [4–6]. Typical clinical signs associated with influenza infection are characterized by an acute onset of fever of short duration, inappetence, lethargy, coughing, dyspnea, and nasal discharge. Morbidity within infected herds is high (approaching 100%), but mortality is typically low (less than 1%) [7]. In recent experimental studies with pandemic influenza (H1N1) 2009 virus, a similar clinical picture has been described [8, 9]. Influenza viruses can also act synergistically with other viral and bacterial pathogens to cause porcine respiratory disease complex [10–12]. The course and severity of an influenza virus infection in pigs are influenced by co-infecting agents, the pig's age, overall health and immune status, and potentially the strain of influenza virus involved [7]. It has been suggested that influenza infections may lead to reduced reproductive performance in affected animals [13]. However, there is insufficient data to conclude that influenza viruses have a specific and direct relationship to the occurrence of reproductive problems in pigs [7].

The favorable health situation provided a unique opportunity to study the clinical impact of infection with pandemic influenza (H1N1) 2009 virus, and this paper describes the results of a case-control study performed on a naïve Norwegian pig subpopulation consisting of all nucleus and multiplier herds.

## 2. Materials and Methods

### 2.1. Study Population and Laboratory Methods

The study population comprised all 118 Norwegian nucleus and multiplier herds, which were all farrow-to-finish herds. The herds were tested serologically for Influenza A specific NP antibodies by ELISA (ID Screen Influenza A Antibody Competition test, IDVET, according to manufacturer's instructions) and for hemagglutinating antibodies using hemagglutination-inhibition (HI) assays according to the method described in the OIE Manual of Diagnostic Tests and Vaccines for Terrestrial Animals [14]. In addition some herds were tested for presence of viral RNA by real-time reverse transcription polymerase chain reaction (rRT-PCR) during the risk period (30th September 2009 until 31st October 2010) [15, 16].

### 2.2. Study Design and Data Collection

The study was designed as a case-control study. A case (positive herd) was defined as a herd with at least one rRT-PCR-positive sample, or if three or more of at least 20 blood samples were positive for antibodies against influenza A virus. If only one or two of the first 20 samples from a herd were positive with ELISA, the herd was retested with blood samples from 20 previously untested pigs and concluded positive if at least one of these samples were positive.

A questionnaire of 137 questions (123 were closed) was created to record demographics, husbandry information on the herds, and variables of interest. All farmers, irrespective of whether they represented case or control herds, were asked to report if they had observed signs of coughing, sneezing, depression, decrease in feed intake, or increase in reproductive disturbances in their pigs. Farmers who reported clinical signs were asked to estimate the proportion of affected pigs in different age groups. In Norway all nucleus and multiplier herds must keep written records (herd health cards) of all treatments irrespective of whether they were performed by a veterinarian or herd personnel. Farmers were asked to review the herd health cards for all veterinary or farmer treatments initiated during the study period.

The animals in the herds were grouped into four age groups: piglets (suckling piglets before weaning at approximately 5 weeks/10 kg), weaners (piglets after weaning, until approximately 30 kg), growers/finishers/recruit sows (from approximately 30 kg to slaughter weight or breeding age/weight), and sows. This age grouping was chosen because this is the way pigs are most commonly grouped and housed in the Norwegian herds. The transition from one group to the next is in most cases synonymous with a change in the pigs environment.

The interviewees were asked to indicate the occurrence of all observed clinical signs. It was emphasized by the interviewer that the occurrence of clinical signs should be reported as a deviation from the herds' normal clinical situation to lessen the risk of attributing regularly occurring clinical signs to the outbreak of pandemic influenza A (2009) virus. Farmers were asked to indicate the severity of observed clinical signs, but difficulties precisely defining degrees of severity between mild, moderate, or severe signs based on farmers subjective observation led to a simplified binomial classification where signs were classified as either present or absent. In addition, they were asked to estimate the duration of clinical signs in the different age groups of animals. The answer alternatives for duration of clinical signs were less than one week, one to two weeks, or more than two weeks.

The questionnaire was distributed by surface mail in the middle of November 2010. A letter was enclosed with the questionnaire encouraging the farmers to familiarize themselves with the questions and informing them that they would be asked to answer the questionnaire in a telephone interview within the following weeks. The interviews were performed by telephone over a period of 7 weeks between November 2010 and January 2011 by the first author. A paper copy of the questionnaire was used to register the answers for each interview. The data collected were later entered into a purpose-built form using Microsoft Excel 2010 (Microsoft Corporation, Redmond, WA, USA). Basic data analyses were performed in this database.

### 2.3. Statistical Methods

Inference statistics were done by calculating the 95% confidence interval (CI) of the binomial proportions, except when numbers were too small for statistical significance, in which case only descriptive statistics are presented.

## 3. Results

**Figure 1:**
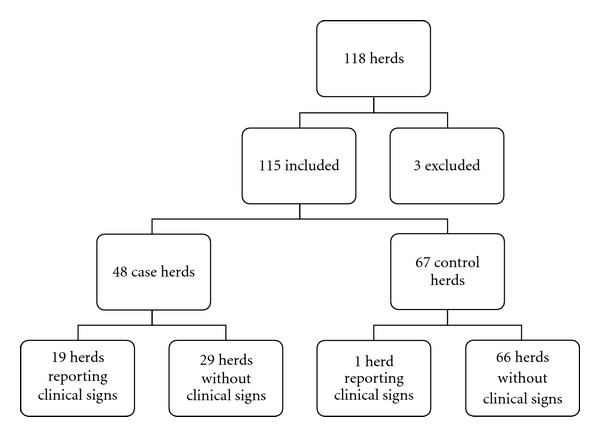
The distribution of the herds in the study population.

All 118 herds answered the questionnaire, giving a response rate of 100%. Three herds were later excluded on the basis of uncertain infection status at the time of the study, leaving the study with 115 herds comprising 47 nucleus herds and 68 multiplier herds. A total of 20 (43%) nucleus herds and 28 (41%) multiplier herds were classified as positive for pandemic influenza (H1N1) 2009 by the case definition. This gave 48 case herds and 67 control herds for the study, which gave a herd prevalence of 42% (95% CI of 33–51%). The distribution of herd categories in the study is shown in Figure 1. In the study population, 100 herds (87%) had batch farrowing, and the distribution of number of weeks between batches was 1 (1 herd), 2 (1 herd), 3 (32 herds), 5.5 (19 herds), and 7 (47 herds). The number of weeks between batches is the period of time elapsed between each time another group of sows is moved to the farrowing unit. The remaining 15 farms practiced continuous farrowing.

Nineteen (40%) (95% CI of 26–55%) of the 48 positive herds reported clinical signs of pig ILI (influenza-like illness) and/or increased reproductive disturbances in one or more age groups. The distributions and the type of observed clinical signs in the different age groups in clinically affected herds are shown in Table 1. Seventeen herds reported clinical signs in sows while 8, 6, and 8 herds reported signs in piglets, weaners, and/or growers/finishers/recruit sows, respectively. With the exception of six herds reporting clinical signs only in sows, and one herd reporting clinical signs only in growers/finishers/recruit sows the remaining 12 herds reported signs in two or more age groups of animals. Three herds reported clinical signs of ILI in all age groups of animals. The proportions of affected animals in the respective age groups are shown in Table 2. Two interviewees were unable to estimate a proportion of affected animals by age group. One of the control herds reported clinical signs of pig ILI as a mild, transient sneezing in approximately 5% of the sows. The remaining 66 (98.5%) negative herds and 29 (60%) positive herds reported no typical disease signs of pig ILI in any age groups.

Clinical signs were reported in similar proportion from all age groups (13–17%), with the exception of decreased feed intake, which was reported with a higher number of observations (25%) in sows. Some farmers also reported fever in weaned piglets, growers/finishers/recruit sows, and sows. Twelve interviewees reported an increase in reproductive disturbances, specifically an increase in returns to estrus, abortions, and decreased litter sizes. Increased numbers of stillbirths were less frequently reported.

The duration of observed clinical signs varied between herds and between age groups. The results for sows are divided in two groups, one group for herds that reported reproductive disturbances and one group for herds that did not report reproductive disturbances. The results of the reported duration in different age groups are shown in Table 3.

## 4. Discussion

This study shows that 40% of positive nucleus and multiplier herds reported clinical signs of pig ILI and/or increased reproductive disturbances. The low morbidity is surprising as one might expect higher morbidity rate given the naïve population and the nature of the disease. The low morbidity, however, corresponds well with another study carried out by the Norwegian Pig Health Service (personal communication Anne Jørgensen) where 51% of infected herds (including non-breeding herds) reported clinical signs. The high health status of pigs in Norway could have resulted in the lower morbidity, as some herds might have experienced subclinical infection or mild disease that was not registered nor reported by the farmer. Farmers were chosen as respondents in this study because they are more likely to have the most complete observations of an influenza outbreak occurring in their farm. While veterinarians are undoubtedly more qualified to perform clinical examinations and evaluations, they normally spend a limited amount of time on each farm, and typically do not observe the animals in a given herd as frequently and regularly as the farmer.

Recall bias is a potential weakness in this retrospective study as the interviews took place approximately one year after the first incursion of pandemic influenza A (2009) virus. Given the Norwegian situation with an outbreak of a previously undiagnosed infectious disease, one would expect farmers to have a heightened awareness and, thus, be more likely to remember and report clinical signs beyond the normal situation. The awareness of pig farmers was also likely affected by the attention given to the outbreak of pandemic influenza A (2009) virus by the public and veterinary health authorities and the media. In addition, the nucleus and multiplier herds in Norway are obliged to keep written records of all treatments of animals, and farmers were encouraged to review these records in a letter enclosed with the questionnaire before the interview. Thus, the high proportion (60%) of positive herds reporting no clinical signs of ILI or increase in reproductive disturbances indicates a high proportion of subclinical infections in cases not complicated by concurrent infections with other respiratory pathogens. The fact that the Norwegian pig population is free from many of the most severe infectious respiratory diseases might lead to a clinical picture less likely to be confounded or masked by concurrent infections. In the present study, only one of the control herds reported clinical signs of ILI.

The low morbidity emphasizes the need for a continued active surveillance program to monitor the status of infection in a naïve pig population. Passive surveillance based on reports of clinical disease would have a low sensitivity as many positive herds would be missed. It also poses challenges when trying to prevent herd-to-herd transmission of pandemic influenza A (2009) virus, as the risks of unintentionally introducing virus-shedding animals to seronegative recipient herds are likely to be increased when the animals are not displaying signs of disease. It also increases the potential risk of pig-to-human transmission. Low morbidity in positive herds indicates a limited economic impact of infection in these herds.

The proportion of infection was approximately the same in both closed, self-replacing nucleus herds and multiplier herds that buy replacement sows from nucleus herds. This supports that introduction of new pigs was unlikely to be the primary source of infection on farms, as previously described by Hofshagen et al. [1].

Information bias is a weakness when open questions are used, especially errors that result from a misunderstanding of questions by respondents. The risk of information bias is, however, reduced in an interview situation by the opportunity to clarify any misunderstandings and by having one person conducting all interviews.

Clinical signs typical of swine influenza were observed in all age groups of animals. Not all infected pigs showed signs even though all were susceptible in the initial phase of infection. Acute outbreaks of swine influenza are more likely to give signs of disease in fully susceptible, seronegative animals. In the present study, the interviews were focused on the alterations in observed signs of disease in the initial phase of the pandemic influenza A (2009) virus outbreak. As described, the Norwegian swine population was free from swine influenza (subtypes H1N1 and H2N3) before the outbreak in 2009 [2]. The observation of clinical signs in all age groups of animals might be the case only in the initial phase of infection in previously naïve herds. When a herd has experienced an infection and subsequent seroconversion, later reintroductions or persistence of infections might lead to clinical signs being observed only in age groups of pigs previously unexposed to active or passive immunization against the specific virus. For instance, the morbidity and duration of clinical signs in piglets and weaners could potentially be affected by maternally derived immunity against influenza [17].

In more than half of the clinically affected herds, decreased feed intake and/or increased reproductive disturbances was reported in sows. These parameters are easily monitored, and farmers use them as reference parameters of performance. As a result, farmers could be more sensitive to alterations in these parameters. None of the control herds reported an increase in reproductive disturbances in sows. The direct role of swine influenza virus in abortions is unclear, and it is commonly believed that the reproductive problems caused by influenza viruses in pigs are due to high fever [18]. Fever was reported in sows, weaners, and growers/finishers/recruit sows, but in this study pyrexia was not emphasized as it was unclear how many farmers routinely checked the rectal temperature of the pigs.

The proportion of observed clinical signs varied between herds and age groups, although the numbers of observations were too small to show statistical significance. This difference could be explained by several factors, the most relevant being herd health status, concurrent infections, differences in sow management, true differences, or recall bias (sows in farrowing unit might “represent” the entire sow population). In addition, some clinical signs (e.g., coughing or sneezing) are more apparent and, therefore, more likely to be recorded and influence the proportion.

In contrast to the low herd morbidity seen in our study, influenza in nonimmune pigs is usually considered to be a disease with high morbidity, low mortality, and with a sudden and remarkable recovery that usually begins within 5–7 days after onset [7]. Experimental infection studies using pandemic influenza (H1N1) 2009 virus have shown a similar clinical picture [8, 9] and reports from natural infections also support this, but with varying morbidity [19, 20]. A recent Australian study in pigs naturally infected with pandemic influenza (H1N1) 2009 virus showed low morbidity and mainly mild clinical signs [21].

This present study show that nearly 50% (95% CI of 29–77%) of the respondents reported a duration of clinical signs of two weeks or more. The reported duration most likely reflects on presented clinical signs within a herd level or epidemiological unit, so one would expect there to be a prolongation because of pig-to-pig transmission after introduction of virus and incubation time. Reproductive disturbances subsequent to an outbreak of ILI will often be observed and recorded for some time after the acute signs have subsided. This was the case in the present study where 88% of recorded clinical signs in sows lasting two weeks or longer were reproductive disturbances. Concurrent or complicating infections, like bacterial infections, can also prolong the clinical manifestation of respiratory illness.

## 5. Conclusion

This study shows the clinical impact of acute infection with pandemic influenza (H1N1) 2009 virus in a naïve pig population. Typical signs of influenza-like illness and/or increased reproductive disturbances were reported from 40% of herds where infection with pandemic influenza (H1N1) 2009 has been documented. Clinical signs were reported from all age groups of animals. The proportion of animals affected, duration, and type of clinical signs varied between herds. Further studies are needed to investigate the reported reproductive disturbances in sows and to evaluate the economic impact of pandemic influenza A (2009) virus infection in the Norwegian pig population.

## Figures and Tables

**Table 1:** Distribution of observed clinical signs in different age groups in clinically positive herds. The number in brackets refers to percentage of herds with these signs in different age groups of pigs.

Clinical signs	Sows (%), *n* = 17	Unweaned piglets (%), *n* = 8	Weaned piglets (%), *n* = 6	Growers, finishers, recruit sows (%), *n* = 8
Coughing	5 (29.4)	5 (62.5)	6 (100)	5 (62.5)
Sneezing	4 (23.5)	4 (50.0)	5 (83.3)	4 (50.0)
Lethargy	7 (41.2)	3 (37.5)	2 (33.3)	2 (25.0)
Fever	6 (35.3)	NR	2 (33.3)	2 (25.0)
Decreased feed intake	12 (70.6)	2 (25.0)	2 (33.4)	4 (50.0)
Abortions	8 (47.0)
Stillbirths	5 (29.4)
Reduced litter sizes	8 (47.0)
Returns to estrus	9 (53.0)

**Table 2:** Proportion of animals showing clinical signs of influenza like various age groups (*n* = number of herds, CI = confidence interval).

	Min. %	Max. %	Mean %	95% CI	
				Lower	Upper
Sows (*n* = 17)	10	100	43.3	28.4	58.2
Piglets (*n* = 8)	10	50	24.6	14.6	34.6
Weaners (*n* = 6)	10	80	41	18.1	63.9
Growers, finishers, recruit sows (*n* = 8)	4	65	27	12.3	41.7

**Table 3:** Duration of clinical signs in herds with reported signs of pig-influenza-like illness.

	Total no. of herds with obs. clinical signs	One week or less	One to two weeks	Two weeks or more	Unsure about duration
Sows	17	6	0	8	3
(i) Reproductive signs	12	2	0	7	3
(ii) Only other clinical signs	5	4	0	1	0
Piglets	8	4	0	4	0
Weaners	6	2	0	2	2
Growers, finishers, recruit sows	8	3	2	2	1
